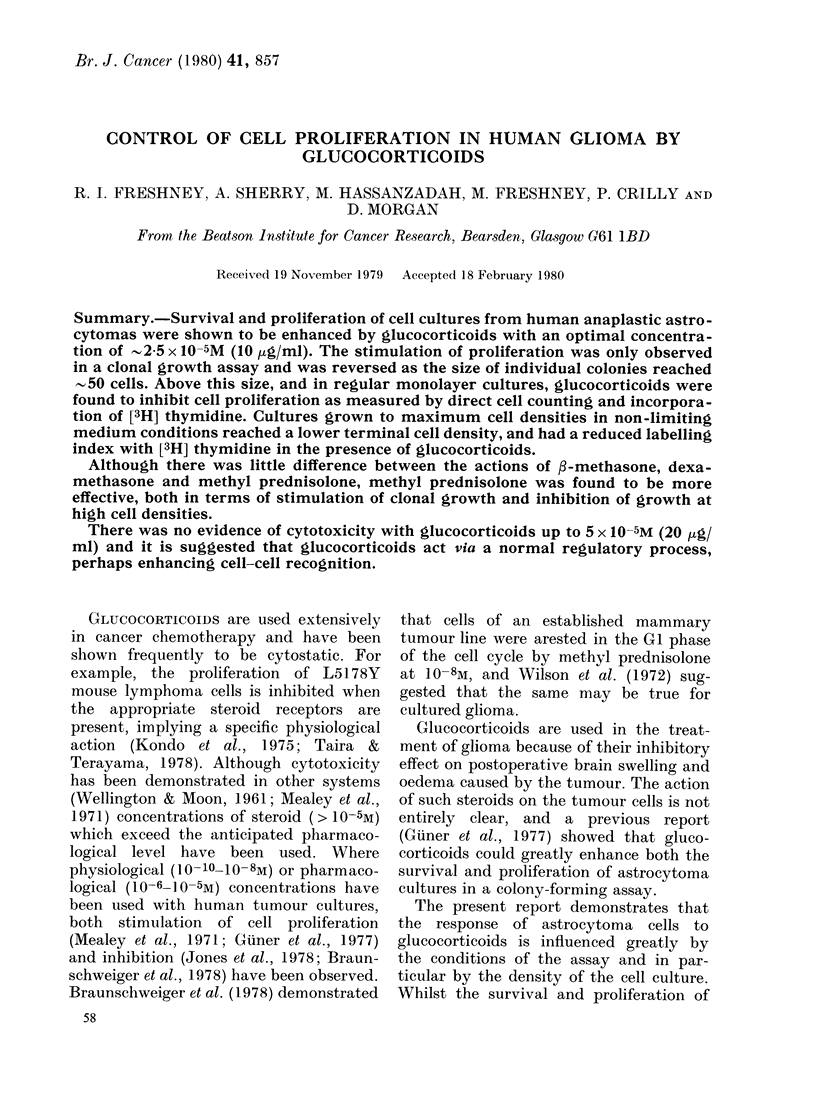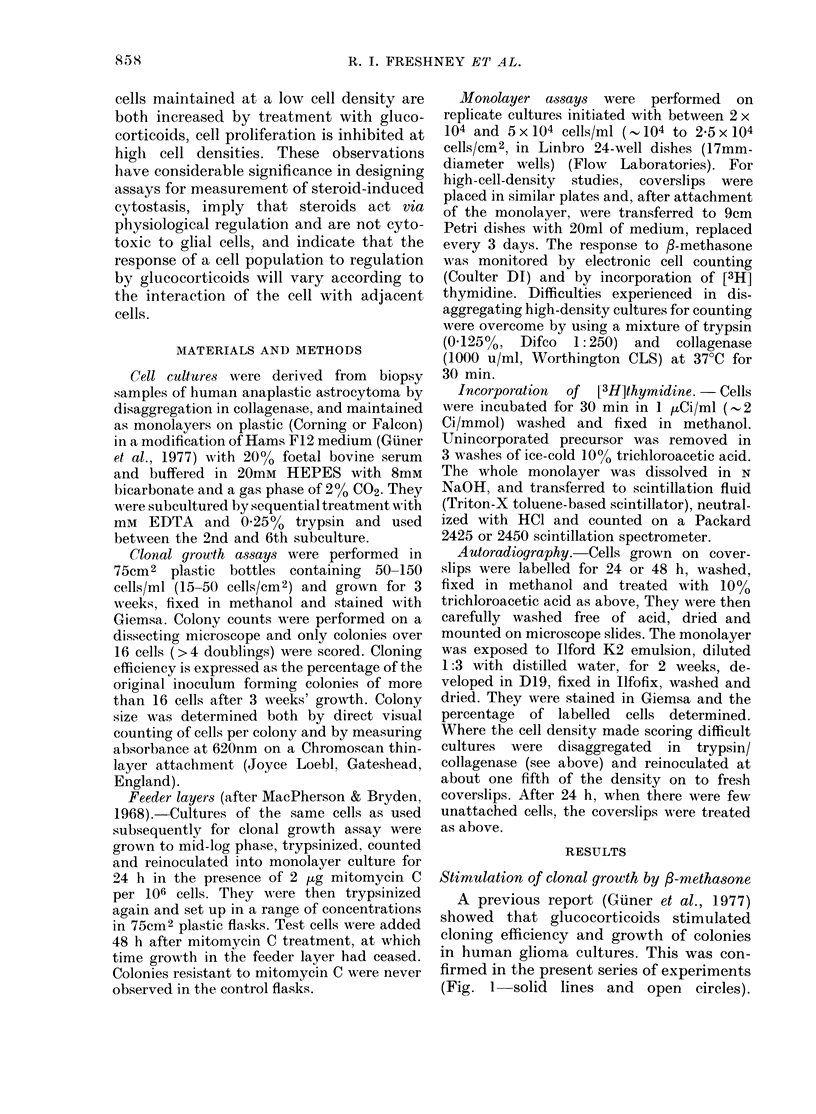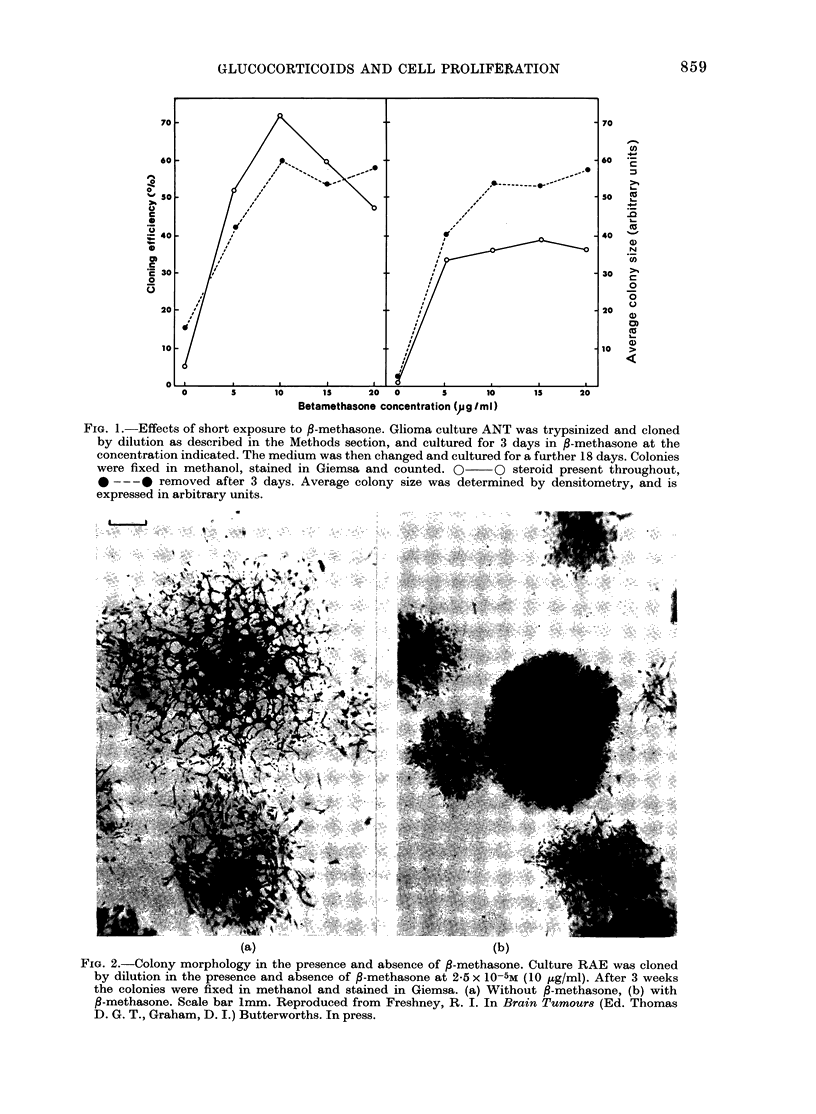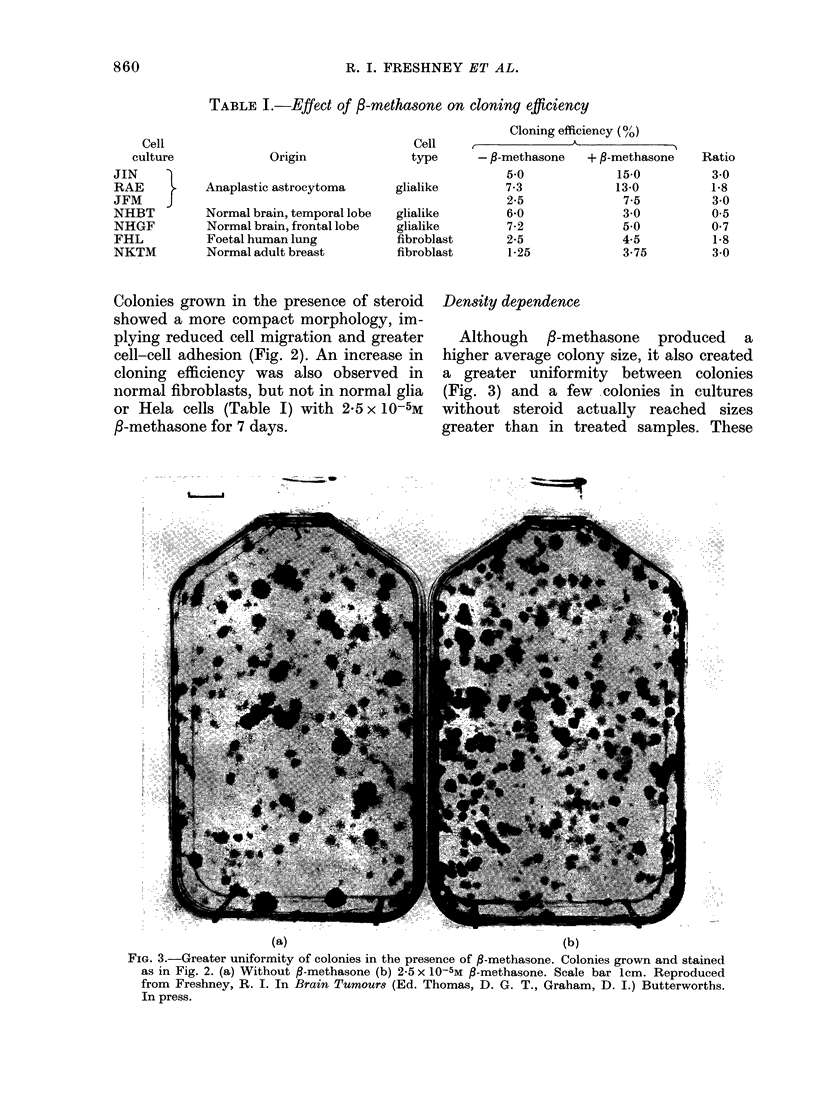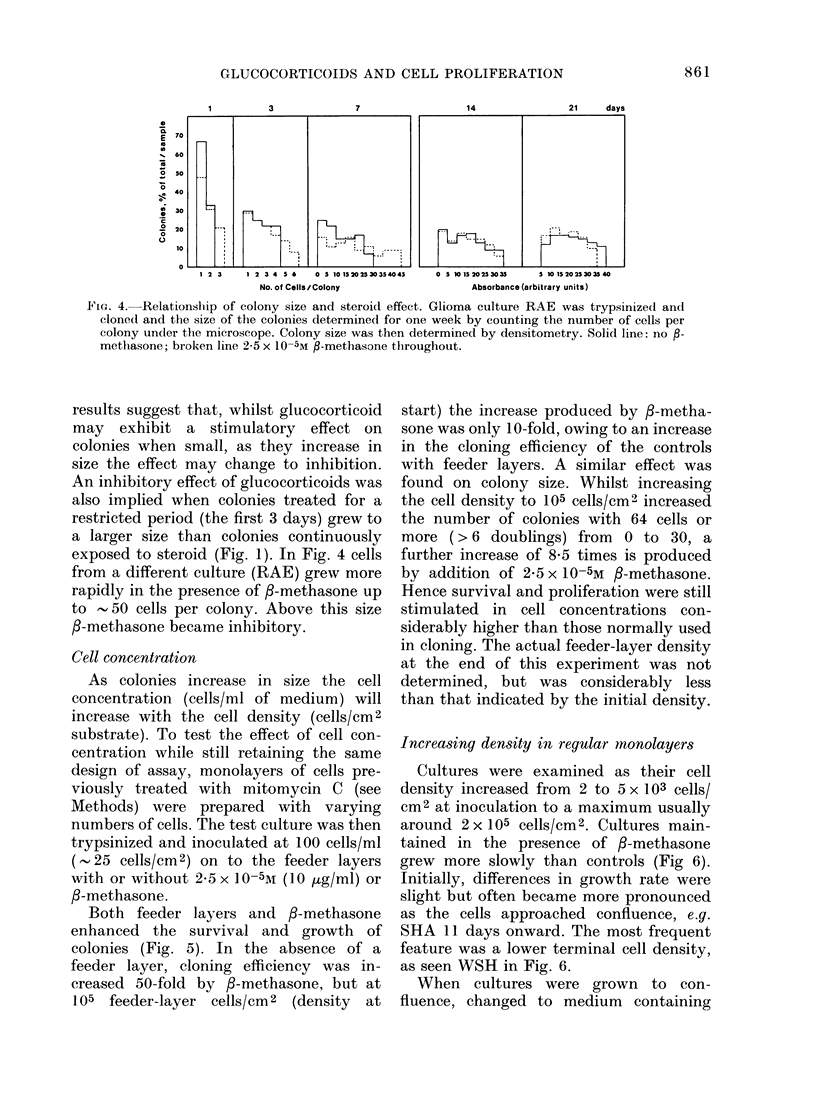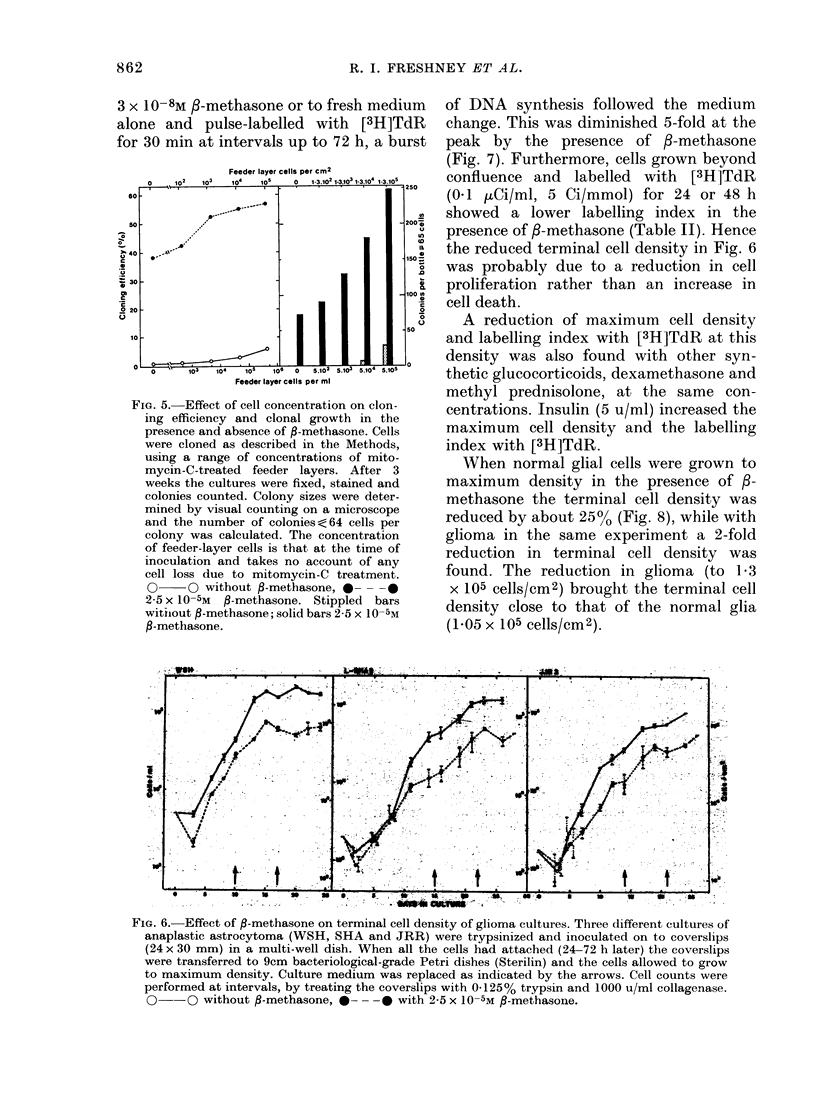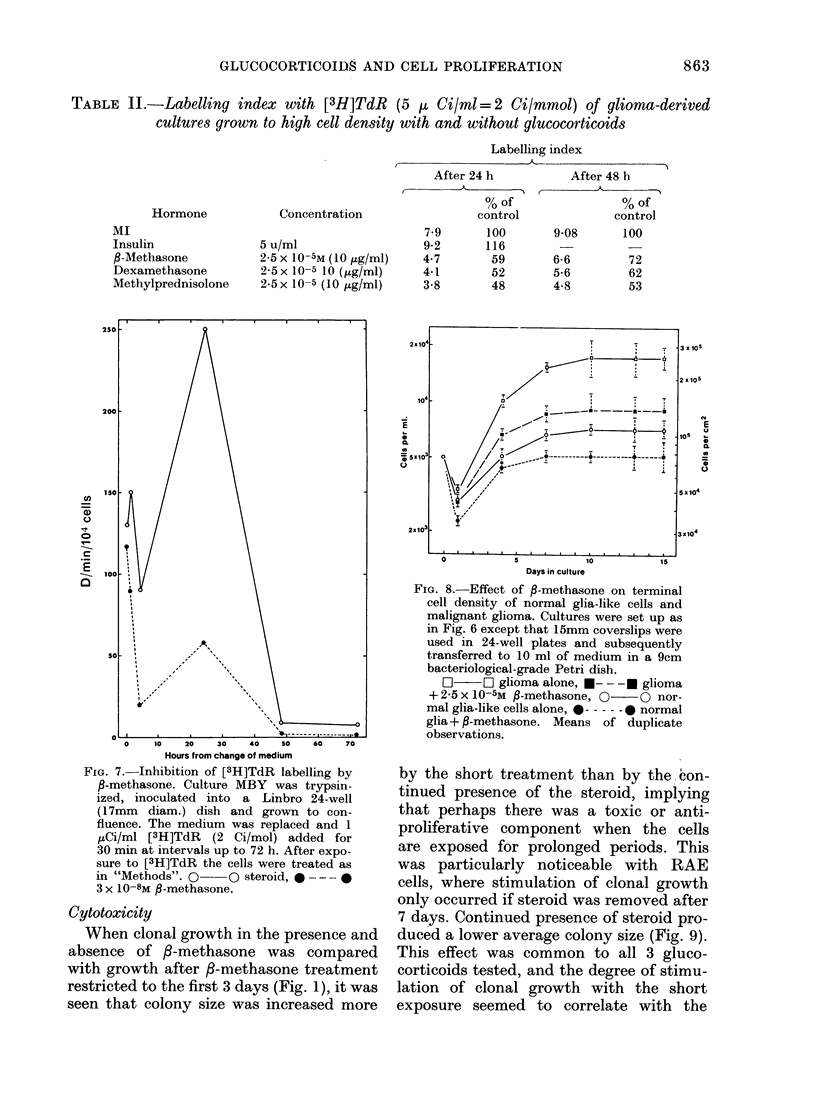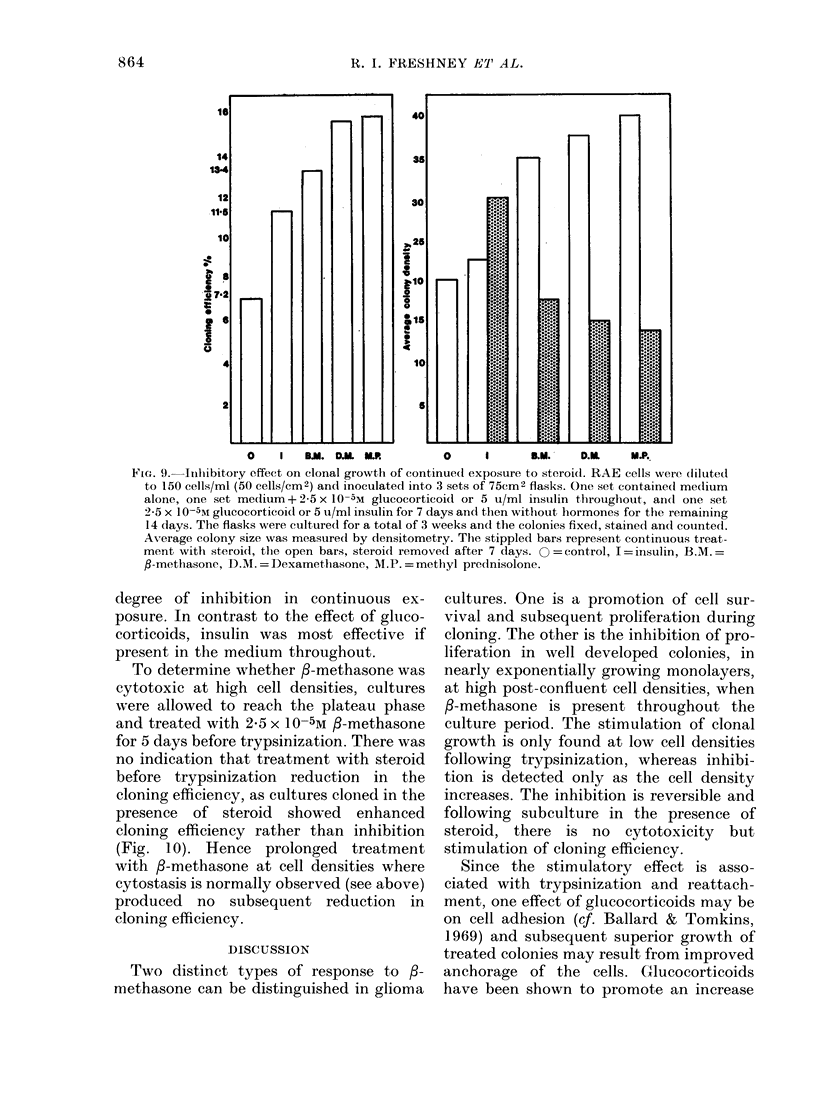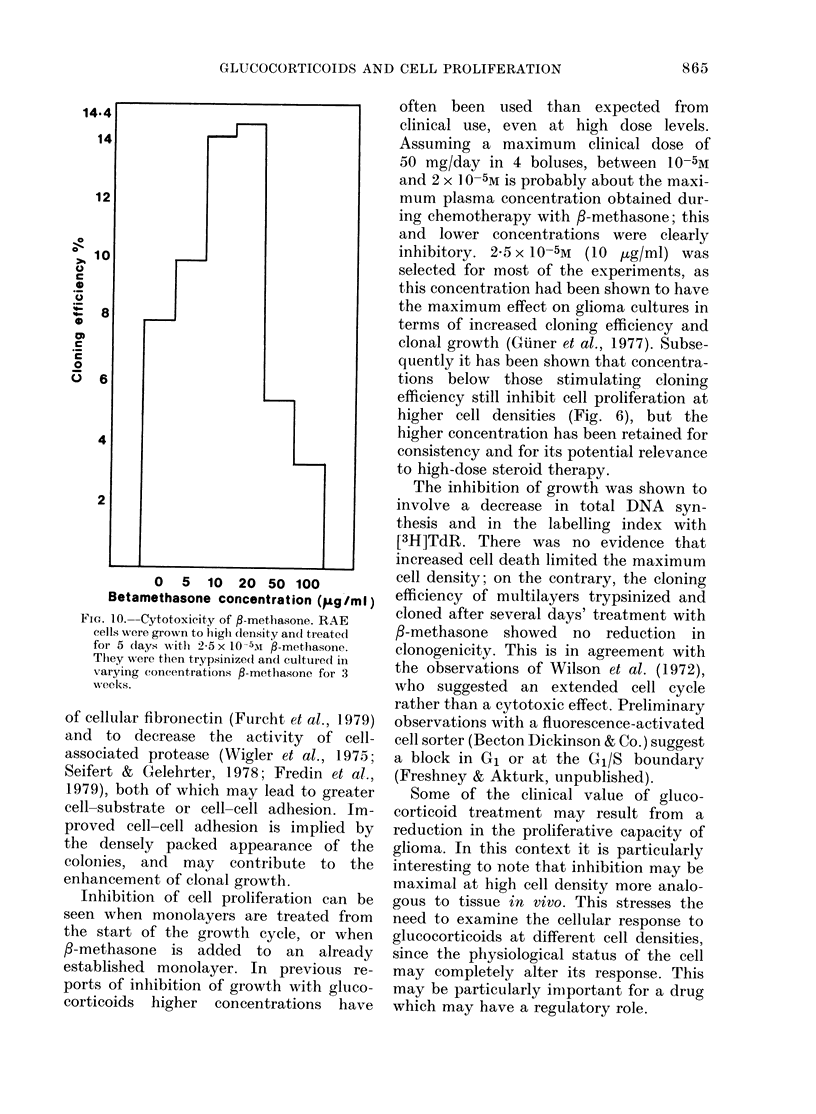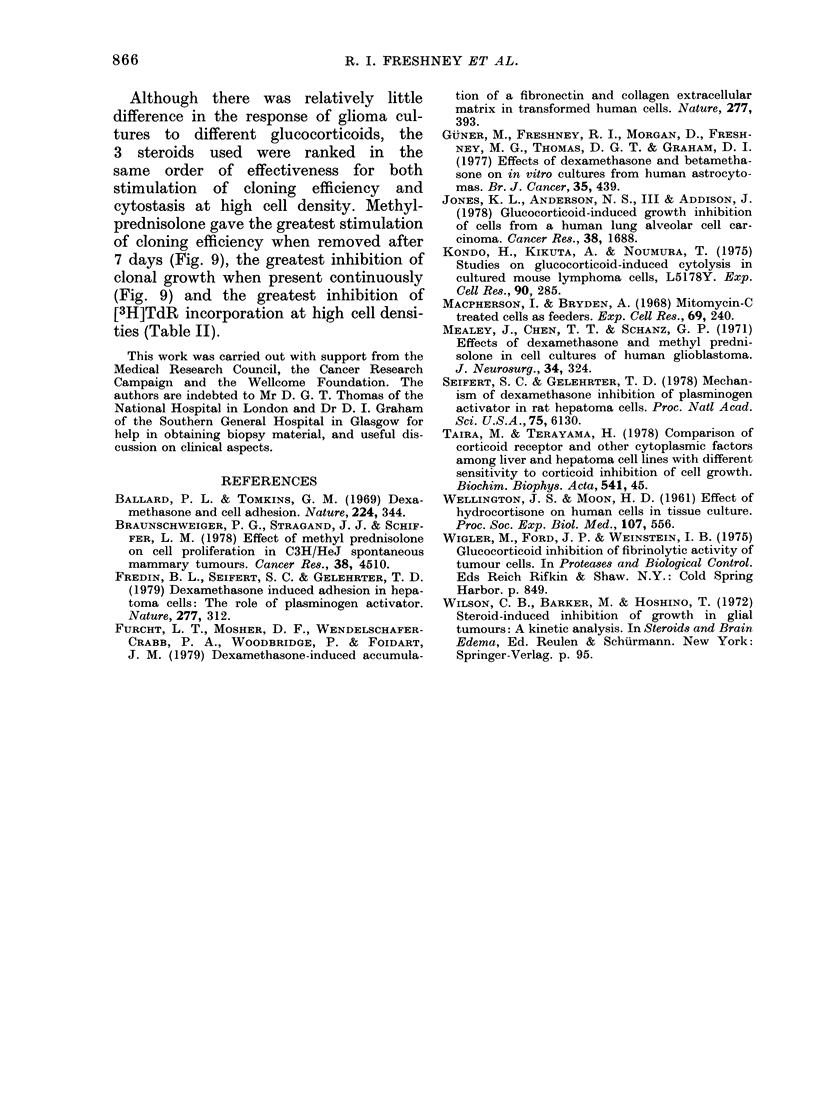# Control of cell proliferation in human glioma by glucocorticoids.

**DOI:** 10.1038/bjc.1980.161

**Published:** 1980-06

**Authors:** R. I. Freshney, A. Sherry, M. Hassanzadah, M. Freshney, P. Crilly, D. Morgan

## Abstract

**Images:**


*Br. J. Cancer* (1980) **41**, 857

### CONTROL OF CELL PROLIFERATION IN HUMAN GLIOMA BY GLUCOCORTICOIDS

R. I. FRESHNEY, A. SHERRY, M. HASSANZADAH, M. FRESHNEY, P. CRILLY AND D. MORGAN

*From the Beatson Institute for Cancer Research, Bearsden, Glasgow G*61 1*BD*

Received 19 November 1979 Accepted 18 February 1980

**Summary.—Survival and proliferation of cell cultures from human anaplastic astrocytomas were shown to be enhanced by glucocorticoids with an optimal concentration of $\sim 2{\cdot}5 \times 10^{-5}$M (10 μg/ml). The stimulation of proliferation was only observed in a clonal growth assay and was reversed as the size of individual colonies reached ~50 cells. Above this size, and in regular monolayer cultures, glucocorticoids were found to inhibit cell proliferation as measured by direct cell counting and incorporation of [$^3$H] thymidine. Cultures grown to maximum cell densities in non-limiting medium conditions reached a lower terminal cell density, and had a reduced labelling index with [$^3$H] thymidine in the presence of glucocorticoids.**

**Although there was little difference between the actions of β-methasone, dexamethasone and methyl prednisolone, methyl prednisolone was found to be more effective, both in terms of stimulation of clonal growth and inhibition of growth at high cell densities.**

**There was no evidence of cytotoxicity with glucocorticoids up to $5 \times 10^{-5}$M (20 μg/ml) and it is suggested that glucocorticoids act *via* a normal regulatory process, perhaps enhancing cell–cell recognition.**

GLUCOCORTICOIDS are used extensively in cancer chemotherapy and have been shown frequently to be cytostatic. For example, the proliferation of L5178Y mouse lymphoma cells is inhibited when the appropriate steroid receptors are present, implying a specific physiological action (Kondo *et al.*, 1975; Taira & Terayama, 1978). Although cytotoxicity has been demonstrated in other systems (Wellington & Moon, 1961; Mealey *et al.*, 1971) concentrations of steroid ($>10^{-5}$M) which exceed the anticipated pharmacological level have been used. Where physiological ($10^{-10}$–$10^{-8}$M) or pharmacological ($10^{-6}$–$10^{-5}$M) concentrations have been used with human tumour cultures, both stimulation of cell proliferation (Mealey *et al.*, 1971; Güner *et al.*, 1977) and inhibition (Jones *et al.*, 1978; Braunschweiger *et al.*, 1978) have been observed. Braunschweiger *et al.* (1978) demonstrated that cells of an established mammary tumour line were arested in the G1 phase of the cell cycle by methyl prednisolone at $10^{-8}$M, and Wilson *et al.* (1972) suggested that the same may be true for cultured glioma.

Glucocorticoids are used in the treatment of glioma because of their inhibitory effect on postoperative brain swelling and oedema caused by the tumour. The action of such steroids on the tumour cells is not entirely clear, and a previous report (Güner *et al.*, 1977) showed that glucocorticoids could greatly enhance both the survival and proliferation of astrocytoma cultures in a colony-forming assay.

The present report demonstrates that the response of astrocytoma cells to glucocorticoids is influenced greatly by the conditions of the assay and in particular by the density of the cell culture. Whilst the survival and proliferation of cells maintained at a low cell density are both increased by treatment with glucocorticoids, cell proliferation is inhibited at high cell densities. These observations have considerable significance in designing assays for measurement of steroid-induced cytostasis, imply that steroids act *via* physiological regulation and are not cytotoxic to glial cells, and indicate that the response of a cell population to regulation by glucocorticoids will vary according to the interaction of the cell with adjacent cells.

### MATERIALS AND METHODS

*Cell cultures* were derived from biopsy samples of human anaplastic astrocytoma by disaggregation in collagenase, and maintained as monolayers on plastic (Corning or Falcon) in a modification of Hams F12 medium (Güner *et al.*, 1977) with 20% foetal bovine serum and buffered in 20mM HEPES with 8mM bicarbonate and a gas phase of 2% $CO_2$. They were subcultured by sequential treatment with mM EDTA and 0·25% trypsin and used between the 2nd and 6th subculture.

*Clonal growth assays* were performed in 75cm$^2$ plastic bottles containing 50–150 cells/ml (15–50 cells/cm$^2$) and grown for 3 weeks, fixed in methanol and stained with Giemsa. Colony counts were performed on a dissecting microscope and only colonies over 16 cells ($>4$ doublings) were scored. Cloning efficiency is expressed as the percentage of the original inoculum forming colonies of more than 16 cells after 3 weeks' growth. Colony size was determined both by direct visual counting of cells per colony and by measuring absorbance at 620nm on a Chromoscan thin-layer attachment (Joyce Loebl, Gateshead, England).

*Feeder layers* (after MacPherson & Bryden, 1968).—Cultures of the same cells as used subsequently for clonal growth assay were grown to mid-log phase, trypsinized, counted and reinoculated into monolayer culture for 24 h in the presence of 2 μg mitomycin C per 10$^6$ cells. They were then trypsinized again and set up in a range of concentrations in 75cm$^2$ plastic flasks. Test cells were added 48 h after mitomycin C treatment, at which time growth in the feeder layer had ceased. Colonies resistant to mitomycin C were never observed in the control flasks.

*Monolayer assays* were performed on replicate cultures initiated with between $2\times10^4$ and $5\times10^4$ cells/ml ($\sim10^4$ to $2{\cdot}5\times10^4$ cells/cm$^2$, in Linbro 24-well dishes (17mm-diameter wells) (Flow Laboratories). For high-cell-density studies, coverslips were placed in similar plates and, after attachment of the monolayer, were transferred to 9cm Petri dishes with 20ml of medium, replaced every 3 days. The response to β-methasone was monitored by electronic cell counting (Coulter DI) and by incorporation of [$^3$H] thymidine. Difficulties experienced in disaggregating high-density cultures for counting were overcome by using a mixture of trypsin (0·125%, Difco 1:250) and collagenase (1000 u/ml, Worthington CLS) at 37°C for 30 min.

*Incorporation of* [$^3$*H*]*thymidine.* — Cells were incubated for 30 min in 1 μCi/ml ($\sim$2 Ci/mmol) washed and fixed in methanol. Unincorporated precursor was removed in 3 washes of ice-cold 10% trichloroacetic acid. The whole monolayer was dissolved in N NaOH, and transferred to scintillation fluid (Triton-X toluene-based scintillator), neutralized with HCl and counted on a Packard 2425 or 2450 scintillation spectrometer.

*Autoradiography.*—Cells grown on coverslips were labelled for 24 or 48 h, washed, fixed in methanol and treated with 10% trichloroacetic acid as above, They were then carefully washed free of acid, dried and mounted on microscope slides. The monolayer was exposed to Ilford K2 emulsion, diluted 1:3 with distilled water, for 2 weeks, developed in D19, fixed in Ilfofix, washed and dried. They were stained in Giemsa and the percentage of labelled cells determined. Where the cell density made scoring difficult cultures were disaggregated in trypsin/collagenase (see above) and reinoculated at about one fifth of the density on to fresh coverslips. After 24 h, when there were few unattached cells, the coverslips were treated as above.

### RESULTS

### *Stimulation of clonal growth by β-methasone*

A previous report (Güner *et al.*, 1977) showed that glucocorticoids stimulated cloning efficiency and growth of colonies in human glioma cultures. This was confirmed in the present series of experiments (Fig. 1—solid lines and open circles).

FIG. 1.—Effects of short exposure to β-methasone. Glioma culture ANT was trypsinized and cloned by dilution as described in the Methods section, and cultured for 3 days in β-methasone at the concentration indicated. The medium was then changed and cultured for a further 18 days. Colonies were fixed in methanol, stained in Giemsa and counted. ○——○ steroid present throughout, ● - - - ● removed after 3 days. Average colony size was determined by densitometry, and is expressed in arbitrary units.

(a) (b)

FIG. 2.—Colony morphology in the presence and absence of β-methasone. Culture RAE was cloned by dilution in the presence and absence of β-methasone at $2{\cdot}5 \times 10^{-5}$M (10 μg/ml). After 3 weeks the colonies were fixed in methanol and stained in Giemsa. (a) Without β-methasone, (b) with β-methasone. Scale bar 1mm. Reproduced from Freshney, R. I. In *Brain Tumours* (Ed. Thomas D. G. T., Graham, D. I.) Butterworths. In press.

TABLE I.—*Effect of β-methasone on cloning efficiency*

| Cell culture | Origin | Cell type | Cloning efficiency (%) − β-methasone | Cloning efficiency (%) + β-methasone | Ratio |
|---|---|---|---|---|---|
| JIN | Anaplastic astrocytoma | glialike | 5·0 | 15·0 | 3·0 |
| RAE | Anaplastic astrocytoma | glialike | 7·3 | 13·0 | 1·8 |
| JFM | Anaplastic astrocytoma | glialike | 2·5 | 7·5 | 3·0 |
| NHBT | Normal brain, temporal lobe | glialike | 6·0 | 3·0 | 0·5 |
| NHGF | Normal brain, frontal lobe | glialike | 7·2 | 5·0 | 0·7 |
| FHL | Foetal human lung | fibroblast | 2·5 | 4·5 | 1·8 |
| NKTM | Normal adult breast | fibroblast | 1·25 | 3·75 | 3·0 |

Colonies grown in the presence of steroid showed a more compact morphology, implying reduced cell migration and greater cell–cell adhesion (Fig. 2). An increase in cloning efficiency was also observed in normal fibroblasts, but not in normal glia or Hela cells (Table I) with $2{\cdot}5 \times 10^{-5}$M β-methasone for 7 days.

*Density dependence*

Although β-methasone produced a higher average colony size, it also created a greater uniformity between colonies (Fig. 3) and a few colonies in cultures without steroid actually reached sizes greater than in treated samples. These

FIG. 3.—Greater uniformity of colonies in the presence of β-methasone. Colonies grown and stained as in Fig. 2. (a) Without β-methasone (b) $2{\cdot}5 \times 10^{-5}$M β-methasone. Scale bar 1cm. Reproduced from Freshney, R. I. In *Brain Tumours* (Ed. Thomas, D. G. T., Graham, D. I.) Butterworths. In press.

FIG. 4.—Relationship of colony size and steroid effect. Glioma culture RAE was trypsinized and cloned and the size of the colonies determined for one week by counting the number of cells per colony under the microscope. Colony size was then determined by densitometry. Solid line: no β-methasone; broken line $2\cdot5 \times 10^{-5}$M β-methasone throughout.

results suggest that, whilst glucocorticoid may exhibit a stimulatory effect on colonies when small, as they increase in size the effect may change to inhibition. An inhibitory effect of glucocorticoids was also implied when colonies treated for a restricted period (the first 3 days) grew to a larger size than colonies continuously exposed to steroid (Fig. 1). In Fig. 4 cells from a different culture (RAE) grew more rapidly in the presence of β-methasone up to ~50 cells per colony. Above this size β-methasone became inhibitory.

*Cell concentration*

As colonies increase in size the cell concentration (cells/ml of medium) will increase with the cell density (cells/cm$^2$ substrate). To test the effect of cell concentration while still retaining the same design of assay, monolayers of cells previously treated with mitomycin C (see Methods) were prepared with varying numbers of cells. The test culture was then trypsinized and inoculated at 100 cells/ml (~25 cells/cm$^2$) on to the feeder layers with or without $2\cdot5 \times 10^{-5}$M (10 μg/ml) or β-methasone.

Both feeder layers and β-methasone enhanced the survival and growth of colonies (Fig. 5). In the absence of a feeder layer, cloning efficiency was increased 50-fold by β-methasone, but at $10^5$ feeder-layer cells/cm$^2$ (density at start) the increase produced by β-methasone was only 10-fold, owing to an increase in the cloning efficiency of the controls with feeder layers. A similar effect was found on colony size. Whilst increasing the cell density to $10^5$ cells/cm$^2$ increased the number of colonies with 64 cells or more (> 6 doublings) from 0 to 30, a further increase of 8·5 times is produced by addition of $2\cdot5 \times 10^{-5}$M β-methasone. Hence survival and proliferation were still stimulated in cell concentrations considerably higher than those normally used in cloning. The actual feeder-layer density at the end of this experiment was not determined, but was considerably less than that indicated by the initial density.

*Increasing density in regular monolayers*

Cultures were examined as their cell density increased from 2 to $5 \times 10^3$ cells/cm$^2$ at inoculation to a maximum usually around $2 \times 10^5$ cells/cm$^2$. Cultures maintained in the presence of β-methasone grew more slowly than controls (Fig 6). Initially, differences in growth rate were slight but often became more pronounced as the cells approached confluence, *e.g.* SHA 11 days onward. The most frequent feature was a lower terminal cell density, as seen WSH in Fig. 6.

When cultures were grown to confluence, changed to medium containing

$3 \times 10^{-8}$M $\beta$-methasone or to fresh medium alone and pulse-labelled with [$^3$H]TdR for 30 min at intervals up to 72 h, a burst of DNA synthesis followed the medium change. This was diminished 5-fold at the peak by the presence of $\beta$-methasone (Fig. 7). Furthermore, cells grown beyond confluence and labelled with [$^3$H]TdR (0·1 $\mu$Ci/ml, 5 Ci/mmol) for 24 or 48 h showed a lower labelling index in the presence of $\beta$-methasone (Table II). Hence the reduced terminal cell density in Fig. 6 was probably due to a reduction in cell proliferation rather than an increase in cell death.

A reduction of maximum cell density and labelling index with [$^3$H]TdR at this density was also found with other synthetic glucocorticoids, dexamethasone and methyl prednisolone, at the same concentrations. Insulin (5 u/ml) increased the maximum cell density and the labelling index with [$^3$H]TdR.

When normal glial cells were grown to maximum density in the presence of $\beta$-methasone the terminal cell density was reduced by about 25% (Fig. 8), while with glioma in the same experiment a 2-fold reduction in terminal cell density was found. The reduction in glioma (to 1·3 $\times 10^5$ cells/cm$^2$) brought the terminal cell density close to that of the normal glia (1·05 $\times 10^5$ cells/cm$^2$).

FIG. 5.—Effect of cell concentration on cloning efficiency and clonal growth in the presence and absence of $\beta$-methasone. Cells were cloned as described in the Methods, using a range of concentrations of mitomycin-C-treated feeder layers. After 3 weeks the cultures were fixed, stained and colonies counted. Colony sizes were determined by visual counting on a microscope and the number of colonies $\leqslant$ 64 cells per colony was calculated. The concentration of feeder-layer cells is that at the time of inoculation and takes no account of any cell loss due to mitomycin-C treatment. ○——○ without $\beta$-methasone, ●- - -● $2{\cdot}5 \times 10^{-5}$M $\beta$-methasone. Stippled bars without $\beta$-methasone; solid bars $2{\cdot}5 \times 10^{-5}$M $\beta$-methasone.

FIG. 6.—Effect of $\beta$-methasone on terminal cell density of glioma cultures. Three different cultures of anaplastic astrocytoma (WSH, SHA and JRR) were trypsinized and inoculated on to coverslips (24 $\times$ 30 mm) in a multi-well dish. When all the cells had attached (24–72 h later) the coverslips were transferred to 9cm bacteriological-grade Petri dishes (Sterilin) and the cells allowed to grow to maximum density. Culture medium was replaced as indicated by the arrows. Cell counts were performed at intervals, by treating the coverslips with 0·125% trypsin and 1000 u/ml collagenase. ○——○ without $\beta$-methasone, ●- - -● with $2{\cdot}5 \times 10^{-5}$M $\beta$-methasone.

TABLE II.—*Labelling index with [³H]TdR (5 μ Ci/ml = 2 Ci/mmol) of glioma-derived cultures grown to high cell density with and without glucocorticoids*

| | | Labelling index | | | |
|---|---|---|---|---|---|
| | | After 24 h | | After 48 h | |
| Hormone | Concentration | | % of control | | % of control |
| MI | | 7·9 | 100 | 9·08 | 100 |
| Insulin | 5 u/ml | 9·2 | 116 | — | — |
| β-Methasone | $2 \cdot 5 \times 10^{-5}$M (10 μg/ml) | 4·7 | 59 | 6·6 | 72 |
| Dexamethasone | $2 \cdot 5 \times 10^{-5}$ 10 (μg/ml) | 4·1 | 52 | 5·6 | 62 |
| Methylprednisolone | $2 \cdot 5 \times 10^{-5}$ (10 μg/ml) | 3·8 | 48 | 4·8 | 53 |

FIG. 7.—Inhibition of [³H]TdR labelling by β-methasone. Culture MBY was trypsinized, inoculated into a Linbro 24-well (17mm diam.) dish and grown to confluence. The medium was replaced and 1 μCi/ml [³H]TdR (2 Ci/mol) added for 30 min at intervals up to 72 h. After exposure to [³H]TdR the cells were treated as in "Methods". ○——○ steroid, ●---● $3 \times 10^{-8}$M β-methasone.

FIG. 8.—Effect of β-methasone on terminal cell density of normal glia-like cells and malignant glioma. Cultures were set up as in Fig. 6 except that 15mm coverslips were used in 24-well plates and subsequently transferred to 10 ml of medium in a 9cm bacteriological-grade Petri dish. □——□ glioma alone, ■---■ glioma + $2 \cdot 5 \times 10^{-5}$M β-methasone, ○——○ normal glia-like cells alone, ●-----● normal glia + β-methasone. Means of duplicate observations.

### *Cytotoxicity*

When clonal growth in the presence and absence of β-methasone was compared with growth after β-methasone treatment restricted to the first 3 days (Fig. 1), it was seen that colony size was increased more by the short treatment than by the continued presence of the steroid, implying that perhaps there was a toxic or antiproliferative component when the cells are exposed for prolonged periods. This was particularly noticeable with RAE cells, where stimulation of clonal growth only occurred if steroid was removed after 7 days. Continued presence of steroid produced a lower average colony size (Fig. 9). This effect was common to all 3 glucocorticoids tested, and the degree of stimulation of clonal growth with the short exposure seemed to correlate with the

FIG. 9.—Inhibitory effect on clonal growth of continued exposure to steroid. RAE cells were diluted to 150 cells/ml (50 cells/$cm^2$) and inoculated into 3 sets of 75$cm^2$ flasks. One set contained medium alone, one set medium + $2{\cdot}5 \times 10^{-5}$M glucocorticoid or 5 u/ml insulin throughout, and one set $2{\cdot}5 \times 10^{-5}$M glucocorticoid or 5 u/ml insulin for 7 days and then without hormones for the remaining 14 days. The flasks were cultured for a total of 3 weeks and the colonies fixed, stained and counted. Average colony size was measured by densitometry. The stippled bars represent continuous treatment with steroid, the open bars, steroid removed after 7 days. ○ = control, I = insulin, B.M. = β-methasone, D.M. = Dexamethasone, M.P. = methyl prednisolone.

degree of inhibition in continuous exposure. In contrast to the effect of glucocorticoids, insulin was most effective if present in the medium throughout.

To determine whether β-methasone was cytotoxic at high cell densities, cultures were allowed to reach the plateau phase and treated with $2{\cdot}5 \times 10^{-5}$M β-methasone for 5 days before trypsinization. There was no indication that treatment with steroid before trypsinization reduction in the cloning efficiency, as cultures cloned in the presence of steroid showed enhanced cloning efficiency rather than inhibition (Fig. 10). Hence prolonged treatment with β-methasone at cell densities where cytostasis is normally observed (see above) produced no subsequent reduction in cloning efficiency.

### DISCUSSION

Two distinct types of response to β-methasone can be distinguished in glioma cultures. One is a promotion of cell survival and subsequent proliferation during cloning. The other is the inhibition of proliferation in well developed colonies, in nearly exponentially growing monolayers, at high post-confluent cell densities, when β-methasone is present throughout the culture period. The stimulation of clonal growth is only found at low cell densities following trypsinization, whereas inhibition is detected only as the cell density increases. The inhibition is reversible and following subculture in the presence of steroid, there is no cytotoxicity but stimulation of cloning efficiency.

Since the stimulatory effect is associated with trypsinization and reattachment, one effect of glucocorticoids may be on cell adhesion (*cf.* Ballard & Tomkins, 1969) and subsequent superior growth of treated colonies may result from improved anchorage of the cells. Glucocorticoids have been shown to promote an increase

FIG. 10.—Cytotoxicity of β-methasone. RAE cells were grown to high density and treated for 5 days with $2{\cdot}5\times10^{-5}$M β-methasone. They were then trypsinized and cultured in varying concentrations β-methasone for 3 weeks.

of cellular fibronectin (Furcht *et al.*, 1979) and to decrease the activity of cell-associated protease (Wigler *et al.*, 1975; Seifert & Gelehrter, 1978; Fredin *et al.*, 1979), both of which may lead to greater cell–substrate or cell–cell adhesion. Improved cell–cell adhesion is implied by the densely packed appearance of the colonies, and may contribute to the enhancement of clonal growth.

Inhibition of cell proliferation can be seen when monolayers are treated from the start of the growth cycle, or when β-methasone is added to an already established monolayer. In previous reports of inhibition of growth with glucocorticoids higher concentrations have often been used than expected from clinical use, even at high dose levels. Assuming a maximum clinical dose of 50 mg/day in 4 boluses, between $10^{-5}$M and $2\times10^{-5}$M is probably about the maximum plasma concentration obtained during chemotherapy with β-methasone; this and lower concentrations were clearly inhibitory. $2{\cdot}5\times10^{-5}$M (10 μg/ml) was selected for most of the experiments, as this concentration had been shown to have the maximum effect on glioma cultures in terms of increased cloning efficiency and clonal growth (Güner *et al.*, 1977). Subsequently it has been shown that concentrations below those stimulating cloning efficiency still inhibit cell proliferation at higher cell densities (Fig. 6), but the higher concentration has been retained for consistency and for its potential relevance to high-dose steroid therapy.

The inhibition of growth was shown to involve a decrease in total DNA synthesis and in the labelling index with [$^3$H]TdR. There was no evidence that increased cell death limited the maximum cell density; on the contrary, the cloning efficiency of multilayers trypsinized and cloned after several days' treatment with β-methasone showed no reduction in clonogenicity. This is in agreement with the observations of Wilson *et al.* (1972), who suggested an extended cell cycle rather than a cytotoxic effect. Preliminary observations with a fluorescence-activated cell sorter (Becton Dickinson & Co.) suggest a block in $G_1$ or at the $G_1$/S boundary (Freshney & Akturk, unpublished).

Some of the clinical value of glucocorticoid treatment may result from a reduction in the proliferative capacity of glioma. In this context it is particularly interesting to note that inhibition may be maximal at high cell density more analogous to tissue *in vivo*. This stresses the need to examine the cellular response to glucocorticoids at different cell densities, since the physiological status of the cell may completely alter its response. This may be particularly important for a drug which may have a regulatory role.

Although there was relatively little difference in the response of glioma cultures to different glucocorticoids, the 3 steroids used were ranked in the same order of effectiveness for both stimulation of cloning efficiency and cytostasis at high cell density. Methylprednisolone gave the greatest stimulation of cloning efficiency when removed after 7 days (Fig. 9), the greatest inhibition of clonal growth when present continuously (Fig. 9) and the greatest inhibition of $[^3H]$TdR incorporation at high cell densities (Table II).

This work was carried out with support from the Medical Research Council, the Cancer Research Campaign and the Wellcome Foundation. The authors are indebted to Mr D. G. T. Thomas of the National Hospital in London and Dr D. I. Graham of the Southern General Hospital in Glasgow for help in obtaining biopsy material, and useful discussion on clinical aspects.

### REFERENCES

BALLARD, P. L. & TOMKINS, G. M. (1969) Dexamethasone and cell adhesion. *Nature*, **224**, 344.

BRAUNSCHWEIGER, P. G., STRAGAND, J. J. & SCHIFFER, L. M. (1978) Effect of methyl prednisolone on cell proliferation in C3H/HeJ spontaneous mammary tumours. *Cancer Res.*, **38**, 4510.

FREDIN, B. L., SEIFERT, S. C. & GELEHRTER, T. D. (1979) Dexamethasone induced adhesion in hepatoma cells: The role of plasminogen activator. *Nature*, **277**, 312.

FURCHT, L. T., MOSHER, D. F., WENDELSCHAFER-CRABB, P. A., WOODBRIDGE, P. & FOIDART, J. M. (1979) Dexamethasone-induced accumulation of a fibronectin and collagen extracellular matrix in transformed human cells. *Nature*, **277**, 393.

GÜNER, M., FRESHNEY, R. I., MORGAN, D., FRESHNEY, M. G., THOMAS, D. G. T. & GRAHAM, D. I. (1977) Effects of dexamethasone and betamethasone on *in vitro* cultures from human astrocytomas. *Br. J. Cancer*, **35**, 439.

JONES, K. L., ANDERSON, N. S., III & ADDISON, J. (1978) Glucocorticoid-induced growth inhibition of cells from a human lung alveolar cell carcinoma. *Cancer Res.*, **38**, 1688.

KONDO, H., KIKUTA, A. & NOUMURA, T. (1975) Studies on glucocorticoid-induced cytolysis in cultured mouse lymphoma cells, L5178Y. *Exp. Cell Res.*, **90**, 285.

MACPHERSON, I. & BRYDEN, A. (1968) Mitomycin-C treated cells as feeders. *Exp. Cell Res.*, **69**, 240.

MEALEY, J., CHEN, T. T. & SCHANZ, G. P. (1971) Effects of dexamethasone and methyl prednisolone in cell cultures of human glioblastoma. *J. Neurosurg.*, **34**, 324.

SEIFERT, S. C. & GELEHRTER, T. D. (1978) Mechanism of dexamethasone inhibition of plasminogen activator in rat hepatoma cells. *Proc. Natl Acad. Sci. U.S.A.*, **75**, 6130.

TAIRA, M. & TERAYAMA, H. (1978) Comparison of corticoid receptor and other cytoplasmic factors among liver and hepatoma cell lines with different sensitivity to corticoid inhibition of cell growth. *Biochim. Biophys. Acta*, **541**, 45.

WELLINGTON, J. S. & MOON, H. D. (1961) Effect of hydrocortisone on human cells in tissue culture. *Proc. Soc. Exp. Biol. Med.*, **107**, 556.

WIGLER, M., FORD, J. P. & WEINSTEIN, I. B. (1975) Glucocorticoid inhibition of fibrinolytic activity of tumour cells. In *Proteases and Biological Control.* Eds Reich Rifkin & Shaw. N.Y.: Cold Spring Harbor. p. 849.

WILSON, C. B., BARKER, M. & HOSHINO, T. (1972) Steroid-induced inhibition of growth in glial tumours: A kinetic analysis. In *Steroids and Brain Edema*, Ed. Reulen & Schürmann. New York: Springer-Verlag. p. 95.